# Recurrent Transpyloric Reflux of a Video Capsule: A Rare Kinetic Sign of Superior Mesenteric Artery Syndrome

**DOI:** 10.1055/a-2884-1505

**Published:** 2026-06-25

**Authors:** Carla Maria Dias De Oliveira, Luis Huaman, Michelina Iafigliola, Casado Casalta, Suhey Perez

**Affiliations:** 1 Gastroenterology and Endoscopy UnitClinic Santa PaulaCaracasMirandaVenezuela, Bolivarian Republic of; 2 Radiology DepartmentClinic Urológico San RománCaracasMirandaVenezuela, Bolivarian Republic of; 3 Gastroenterology UnitClinic ChilemexPuerto OrdazBolívarVenezuela, Bolivarian Republic of; 4 Radiology UnitHelitac GuayanaPuerto OrdazBolivarVenezuela, Bolivarian Republic of; 5 Department of ChemistryUniversidad MetropolitanaCaracasMirandaVenezuela, Bolivarian Republic of

## A case description



**Video 1** Video capsule endoscopy demonstrating the ‘to-and-fro’ motion and recurrent transpyloric reflux. The clip shows the capsule repeatedly failing to progress through the third portion of the duodenum and returning to the gastric cavity on six separate occasions, a characteristic kinetic sign of superior mesenteric artery syndrome.


**Fig. 1:**
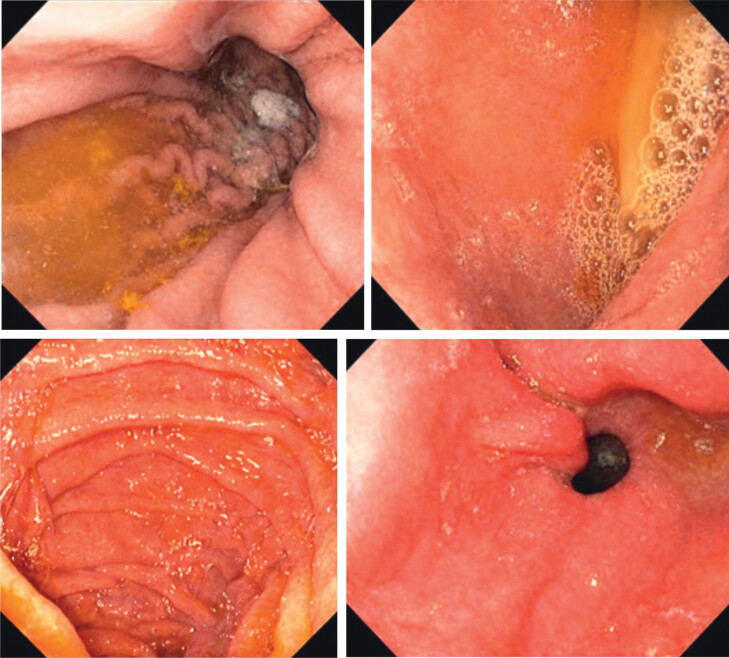
Video gastroscopy revealing significant biliary reflux and signs of enterogastric reflux gastritis (Sydney classification).

**Fig. 2:**
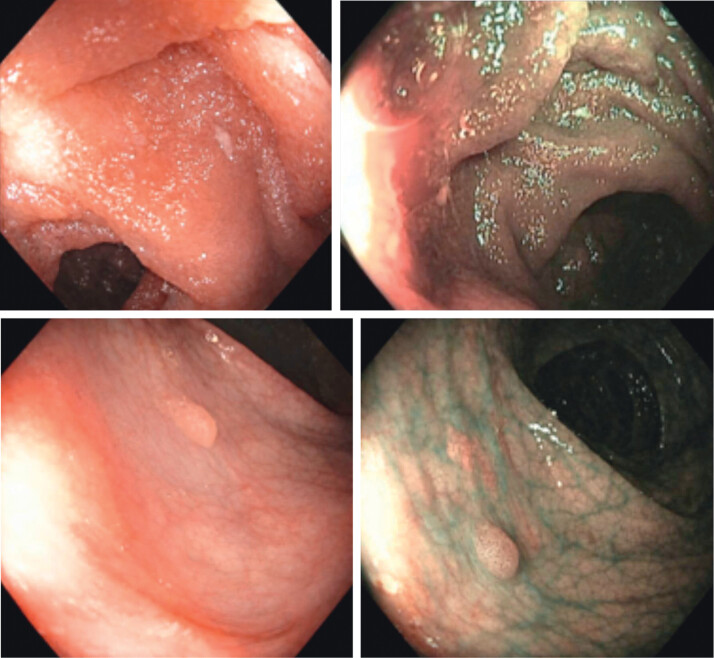
Video ileocolonoscopy showing an ulcerative-like lesion in the terminal ileum and a 0-Is lesion (Paris classification) in the transverse colon.

**Fig. 3:**
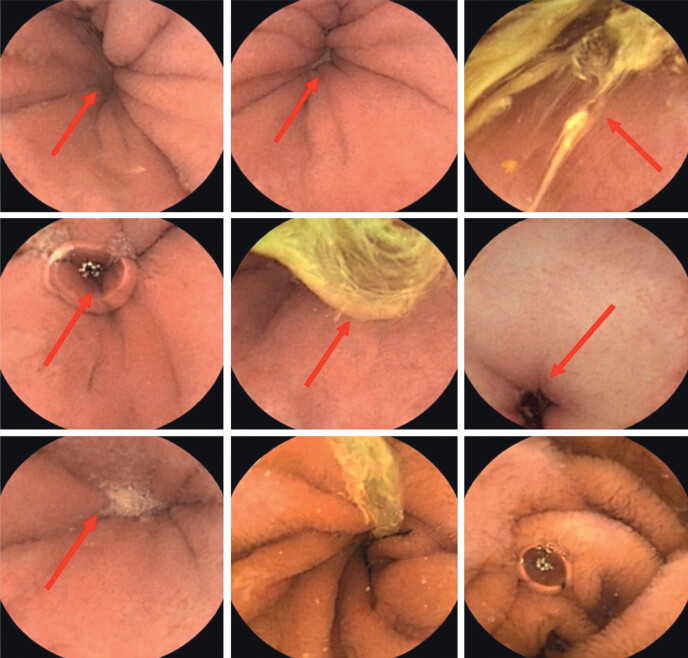
A video capsule endoscopy (VCE) still image capturing the duodenal entrapment and the abundant biliary fluid present in the distal duodenum prior to the transpyloric rebound.

**Fig. 4:**
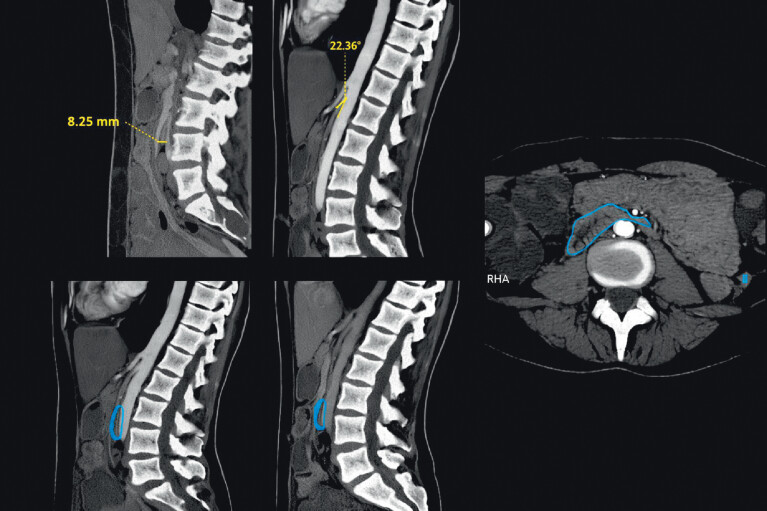
CT enterography demonstrating extrinsic compression of the third portion of the duodenum (D3) by the superior mesenteric artery, resulting in luminal narrowing and proximal stasis.

**Fig. 5:**
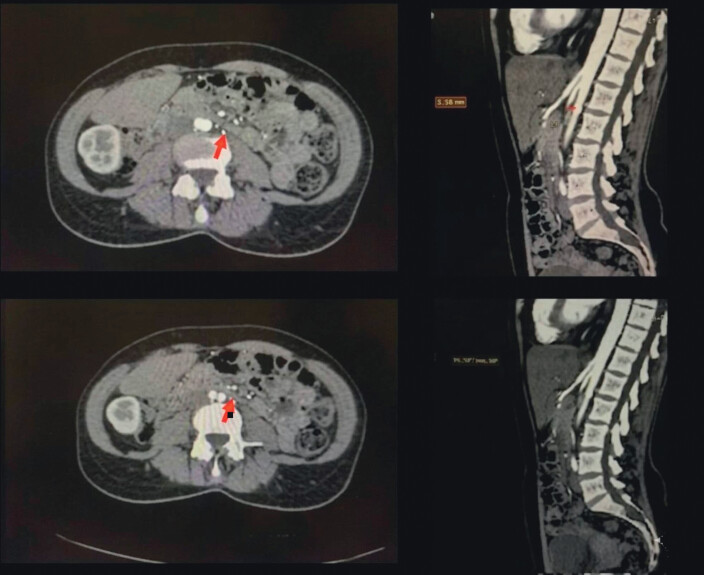
CT angiography confirming an aortomesenteric angle of 14.7° and an aortomesenteric distance of 5.36 mm, with an absence of mesenteric fat, diagnostic of superior mesenteric artery syndrome.

Superior mesenteric artery (SMA) syndrome, also known as Wilkie’s syndrome, is a rare cause of duodenal obstruction resulting from compression of the third portion of the duodenum between the SMA and the abdominal aorta. While diagnosis is typically confirmed via cross-sectional imaging, video capsule endoscopy (VCE) can provide unique dynamic insights into the functional impact of the obstruction. 1​ 2​ 3​ ​4​ A 52-year-old woman presented to our gastroenterology clinic with a chronic history of abdominal pain, bloating, and a significant 10-kg weight loss. She reported an alternating bowel pattern (Bristol Scale Type 4 and 5) associated with meteorism, flatulence, and persistent fatigue. Laboratory tests for celiac disease and food allergies were within normal limits; however, a stool analysis revealed Grade III Dysbiosis. Under anesthesiology monitoring, a bidirectional endoscopy was performed. Video gastroscopy revealed significant biliary reflux and enterogastric reflux gastritis ( Fig. 1 ), while video ileocolonoscopy showed findings suggestive of ulcerative enteropathy in the terminal ileum and a 0-Is lesion (Paris Classification) in the transverse colon ( Fig. 2 ). Histopathology reported reactive antral gastropathy, mild non-specific chronic duodenitis, and focal active colitis. Polyp: Tubular adenoma, negative for high-grade dysplasia. Given the persistence of symptoms and the non-specific nature of the histological findings which did not fully explain the severe biliary reflux and weight loss a VCE was scheduled. The procedure revealed a highly unusual kinetic pattern: a ‘to-and-fro’ motion within the duodenum. Starting at 9.99 minutes of duodenal transit, the capsule exhibited significant progression failure, returning to the stomach on six separate occasions (at 0.11, 1.04, 7.03, 59.38, 74.49, and 119.36 minutes post-duodenal entry). At 131.36 minutes, the final re-entry into the duodenum occurred, where the capsule remained stationary until 195.13 minutes ( Video 1 ). No mucosal lesions were observed, although abundant biliary fluid was present ( Fig. 3 ). Subsequent computed tomography (CT) enterography revealed extrinsic compression of the third portion of the duodenum ( Fig. 4 ), and CT angiography confirmed an aortomesenteric angle of 14.7° and a distance of 5.36 mm ( Fig. 5 ). This mechanical barrier explains both the biliary reflux and the dysbiosis caused by chronic slowing of duodenal transit. This case highlights a novel endoscopic finding for SMA syndrome: the recurrent transpyloric reflux of the video capsule. This ‘rebound’ phenomenon serves as a key diagnostic clue for extrinsic mechanical obstruction, and VCE offers valuable functional data regarding the severity of duodenal stasis in patients with suspected Wilkie’s syndrome.

Endoscopy_UCTN_Code_CCL_1AB_2AH_3AB
